# Exploring the binding sites of VRT534 at Cx26 as a putative chemical chaperone for targeted treatment of hereditary hearing disorders

**DOI:** 10.3389/fmed.2025.1607598

**Published:** 2025-09-24

**Authors:** Jennifer Harre, Dahua Wang, Athanasia Warnecke, Carsten Zeilinger

**Affiliations:** ^1^Department of Otorhinolaryngology, Head- and Neck-Surgery, Hannover Medical School (MHH), Hannover, Germany; ^2^Cluster of Excellence EXC1077 “Hearing4all”, German Research Foundation (DFG; “Deutsche Forschungsgemeinschaft”), Hannover, Germany; ^3^Gottfried-Wilhelm-Leibniz University of Hannover, BMWZ (Zentrum für Biomolekulare Wirkstoffe), Hannover, Germany

**Keywords:** hearing loss, connexin26, syncro-patch, chemical chaperone, Diff-Dock-L, VRT534

## Abstract

Hearing loss is the most common sensory disorder, significantly affecting the quality of life for millions of people worldwide. Chemical chaperones are emerging as a potential therapeutic option for hereditary forms of deafness associated with protein misfolding. VRT534, a chemical chaperone previously used in the treatment of cystic fibrosis, has been shown to modulate the activity of mutated forms of human connexin 26 (Cx26), a gap junction protein crucial for auditory function. However, the precise molecular mechanism of its interaction with Cx26 remains unclear. In this study, we investigated the ability of VRT534 to bind and functionally rescue mutant Cx26. Structural models of Cx26 were generated using AlphaFold3 and analyzed via Diff-Dock-L for binding prediction. Functional restoration by VRT534 was tested using an automated patch-clamp in HeLa cells expressing wild-type or mutant Cx26 (Cx26WT, Cx26L90P, Cx26F161S, and Cx26R184P). VRT534 restored channel function in Cx26L90P and Cx26R184P, but not in Cx26F161S. Docking data revealed stronger binding affinity of VRT534 to mutant variants, with putative binding sites located near the pore region. These findings provide new insight into the selective rescue of mutant Cx26 and support further development of chemical chaperones for hereditary hearing loss.

## 1 Introduction

Hearing loss is a major global health challenge, affecting over 7% of the world's population. Despite technological advances in hearing aids and cochlear implants, therapeutic strategies addressing the molecular basis of hereditary hearing loss remain lacking (1). Genetic factors account for more than half of congenital hearing loss cases (2–4), with mutations in the GJB2 gene, which encodes connexin 26 (Cx26), being the most prevalent cause of non-syndromic sensorineural hearing loss (5).

Connexins are a family of transmembrane proteins that form the structural subunits of gap junctions. Each connexin has four transmembrane domains, two extracellular loops, one intracellular loop, and intracellular N- and C-termini. Different connexin types can combine to form diverse gap junction channels, allowing for cell-type-specific communication. Connexin 26 (Cx26) and connexin 43 (Cx43) are among the most studied. A connexon, also known as a hemichannel, is a hexameric assembly of six connexin proteins. Connexons are embedded in the plasma membrane and represent half of a gap junction channel. Each connexon can exist in an open or closed state and can function as an independent hemichannel or form a complete intercellular channel by docking with another connexon from an adjacent cell. The formation of a gap junction requires the alignment and docking of two connexons from neighboring cells, creating a continuous aqueous pore. Connexon hemichannels and gap junctions facilitate the exchange of ions, metabolites, and signaling molecules (6). Gap junction proteins are essential for potassium recycling in the cochlea, maintaining the endocochlear potential critical for auditory transduction. Mutations in Cx26 disrupt the formation of functional gap junctions, leading to cellular dysfunction and hearing loss, while preserving the cochlear architecture (7–14). This disruption offers a therapeutic target: restoring gap junction communication through the use of chemical chaperones.

The potential for small-molecule chaperones to stabilize mutant Cx26 was recently described by Wang et al. (15). This strategy has been successfully used in the treatment of cystic fibrosis and other diseases (16–18). More than 100 disease-associated Cx26 mutations have been described, many of which led to protein misfolding, degradation, or impaired trafficking (19, 20). Advances in structural biology, particularly through cryo-electron microscopy, have revealed how specific mutations disrupt Cx26 architecture and function (21–23). Among these, the mutations (Cx26L90P, Cx26F161S, and Cx26R184P) impair channel function to varying degrees (24, 25). To explore the mechanism of VRT534-mediated rescue, we modeled mutant and wild-type Cx26 structures and examined functional recovery using patch-clamp analysis in transfected HeLa cells.

## 2 Materials and methods

### 2.1 Cell cultivation and harvesting

HeLa cells expressing either wild-type Cx26 (Cx26WT) or mutantsCx26L90P, Cx26F161S, Cx26R184P were cultivated at 37°C with 5% CO_2_. After 3 days, cells were detached using TypLE solution, washed with PBS, and resuspended in extracellular solution. Cells were triturated and diluted in extracellular solution and cooled in a low-binding Petri dish for 15 min at ~10°C. The cell suspension was transferred to a SyncroPatch Teflon reservoir and placed on the Cell Hotel set to 10°C with 200 RPM shaking frequency. No explicit seal resistance threshold was used during the analysis; however, recordings with seal resistance below 500 MΩ were excluded to ensure data quality. Cell viability post-harvesting was confirmed by trypan blue exclusion (>95%).

### 2.2 Patch-clamp analysis

Automated patch-clamp recordings were conducted using the SyncroPatch 384i platform (Nanion Technologies). Medium (5–8 MΩ) and high resistance (≥10 MΩ) chips were used. Current–voltage relationships were measured using a step protocol from −80 mV to +60 mV in 5 mV increments. For activation, cells were depolarized to +40 mV for 10 or 20 s and hyperpolarized to −100 or −120 mV for 30 or 60 s. Higher voltages (~+80 mV) were avoided due to stability issues in prolonged recordings. Steady-state activation and inactivation curves were fitted with Boltzmann functions. Compound effects were normalized to baseline and maximum block, and IC50 values were calculated using GraphPad Prism (version 6.05).

### 2.3 Structural modeling and docking

3D models of human Cx26WT and its mutants were generated using AlphaFold3 based on DNA sequences (26, 27). Only monomeric forms were modeled, as the Diff-Dock-L algorithm currently supports docking to monomers. Full connexon modeling would require molecular dynamic simulations (MDSs), which were beyond the scope of this study. Structural models were validated by alignment with available cryo-EM structures. Docking simulations with VRT534 were performed using Diff-Dock-L, and binding scores were reported in kcal/mol. Lower (more negative) scores indicate stronger binding affinity.

## 3 Results

**Figure 1:**
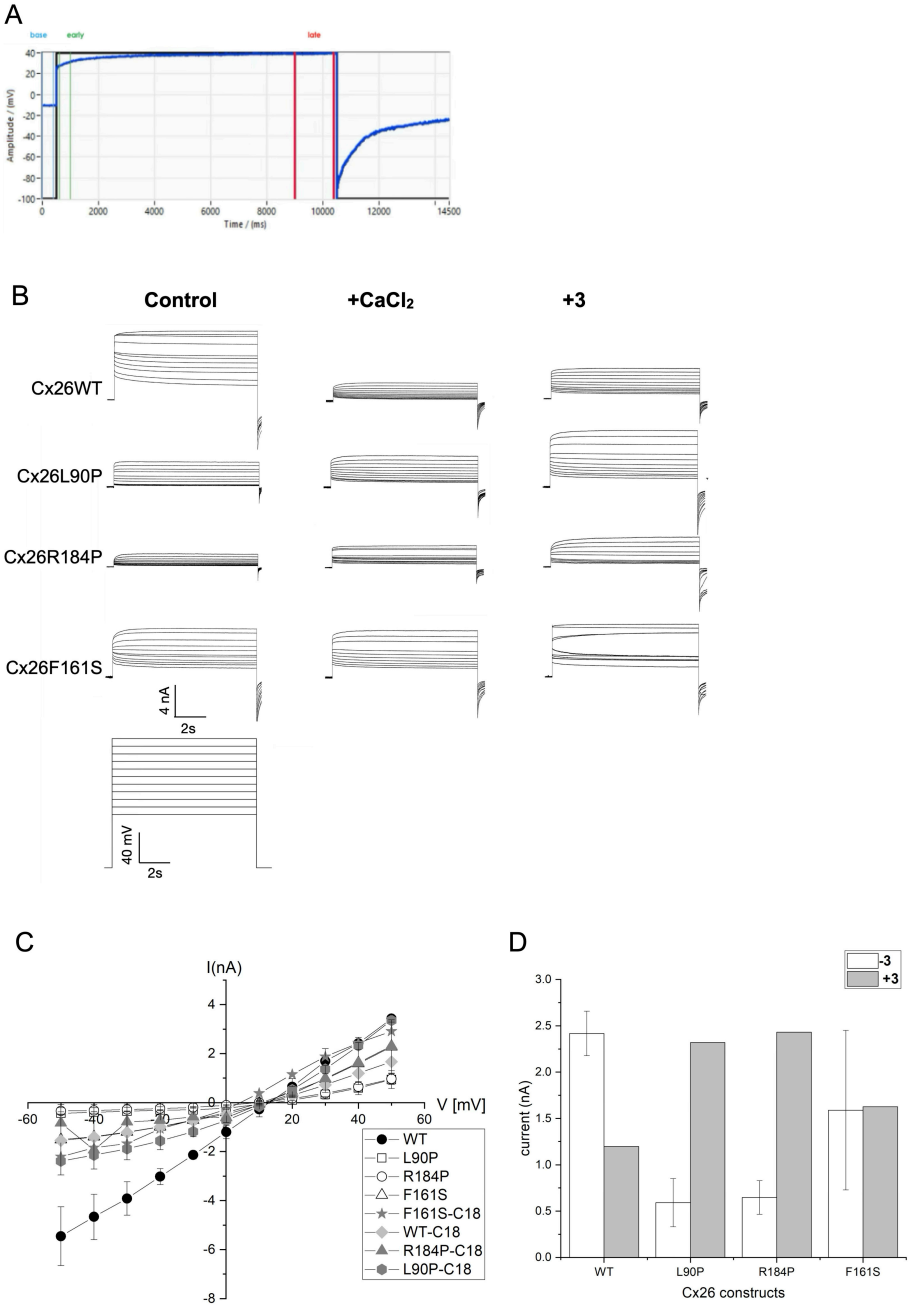
**(A)** Automated voltage protocol as a function of time. **(B)** Typical current traces after applying voltages for HeLa cells expressing Cx26WT, or mutant Cx26F161S, Cx26L90P, or Cx26R184P in the presence of low or normal Ca^2+^ or preincubated with VRT534 (20 μM). **(C)** I–V plot obtained from all constructs Cx26WT, Cx26F161S, Cx26L90P, and Cx26R184P with and without VRT534. **(D)** Channel activity restoration by VRT534 treatment. Current measurements at +40 mV for all constructs: Cx26WT, Cx26F161S, Cx26L90P, and Cx26R184P.

**Figure 2:**
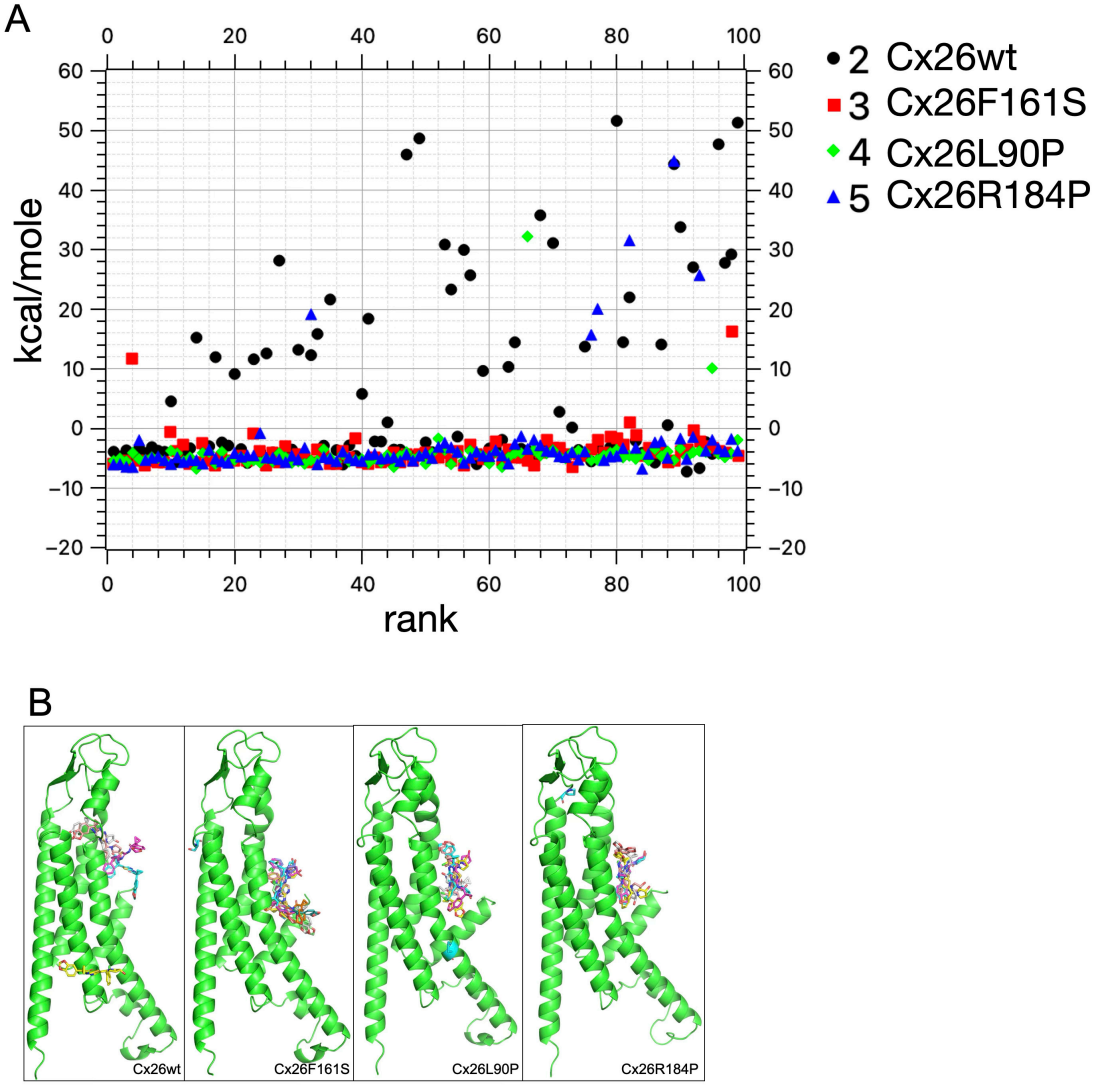
Human Cx26WT and mutations modeled by AlphaFold3 for docking experiments. **(A)** Cx26 DNA sequence was used for AlphaFold3 modeling. The monomeric Cx26 models for wild-type and mutations in Cx26F161S, Cx26L90P, and Cx26R184P, shown as a cartoon (green), were transferred to Diff-Dock-L for docking experiments. Diff-Dock-L analysis of putative binders of VRT534 using rank as a function of SMINA affinity in kcal/mole for Cx26WT and mutant positions Cx26F161S, Cx26L90P, and Cx26R184P (32–36). **(B)** Binding position with an affinity greater than −6 kcal/mol of VRT534 in the structure of monomeric human Cx26 (green) with several positions for VRT534 near the internal pore.

To investigate whether VRT534 influences the connexin 26 wild-type or mutant activities, HeLa cells expressing Cx26WT or the mutant constructs Cx26F161S, Cx26L90P, and Cx26R184P were used. Therefore, the cells were equilibrated in a cell hotel at different conditions) and then analyzed using the automated SyncroPatch-clamp process, analyzing the Cx26-mediated channel activity (Table 1). Sealing and voltage testing were performed as shown in Figure 1A. Automated patch-clamp analysis revealed that HeLa cells expressing Cx26L90P, Cx26F161S, or Cx26R184P showed reduced channel activity compared to Cx26WT (Figure 1B). The obtained current traces were monitored as a function of voltage with or without high Ca^2+^ (5 mM) or 20 μM VRT534. The control shows that Cx26 mutants exhibited lower channel activity, while amino acid exchange at position F161S displayed diminished Ca^2+^ sensitivity, suggesting altered gating properties. After overnight preincubation with 20 μM VRT534, current traces were plotted as a function of voltage for control and VRT534 treatment in Cx26WT and Cx26 mutant cells (Figure 1C). The influence on the restoration of Cx26WT and mutant HeLa cells was monitored at + 40 mV, and it can be seen that the best recovery effect was achieved for Cx26 mutant L90P and Cx26R184P (Figures 1C, D). To investigate whether a structural position can be detected for VRT534, the DNA sequence of human Cx26 was modeled via AlphaFold3, and the structure of the monomeric Cx26 was used for docking experiments. The binding affinities of 100 binding positions were determined for each monomeric structure of Cx26WT and the mutated Cx26F161S, Cx26L90P, and Cx26R184P (Figure 2A). For Cx26WT, binding affinities were predominantly weak (positive or near-zero kcal/mol). In contrast, Cx26L90P and Cx26R184P showed multiple high-affinity ( ≤ -6 kcal/mol) binding positions near the pore region (Figure 2B). Interestingly, these positions were not adjacent to the mutated residues, suggesting an allosteric mechanism of action. The lack of rescue in Cx26F161S, despite pore-proximal binding, suggests that not all gating defects are reversible by VRT534. This may reflect a mechanistic divergence between Ca^2+^-sensitive gating and chaperone-mediated structural stabilization.

**Table 1:** Cx26-mediated channel activity by automated SyncroPatch-Clamp analysis.

**Chip**	**Ca^2+^-condition**	**Cx26-constructs**	**CBN (concentrations in the cell hotel)**
Chip#1	Low Ca^2+^	WT, L90P, R184P, F161S	No CBN, 2 μM, 20 μM
Chip#2	Normal Ca^2+^	WT, L90P, R184P, F161S	No CBN, 2 μM, 20 μM
Chip#3	Low & Normal Ca^2+^	WT, L90P, R184P, F161S	160 μM CBN

Conditions of channel activity using Ca^2+^ and carbenoxolone (CBN) as control and different VRT534 concentrations during preincubation in the cell hotel. The cell line assignment varies, but all four variants (WT, L90P, R184P, and F161S) were tested under all conditions. Positions B4/C7/C9, etc., denote well IDs on the patch chip.

## 4 Discussion

This study introduces a combined structural and functional approach to investigating chemical chaperone action in mutated Cx26 channels. Our data support the hypothesis that VRT534 selectively restores function by binding near the pore region, independent of the exact mutation site. Despite promising research, implementing chemical chaperone therapy for hearing loss faces significant barriers. These include ensuring action specificity and the reliable delivery of chaperones to cochlear cells, given the restrictive nature of the cochlear environment. Structure-based approaches allow for the precise design of chemical chaperones. In addition, the used mutations have been shown to have different effects on function: they can prevent the protein from reaching the cell membrane or cause it to function incorrectly. Chemical chaperones may need to be probed for these different effects. Recently, we used a microarray test to identify the chemical chaperone VRT534, which restores the Cx26 mutation-induced malfunction of connexin hemichannels in HeLa cells (7). This could indicate that chemical chaperones favor channel opening, or, as shown in cystic fibrosis, influence the folding process. For the first time to our knowledge, we used the SyncroPatch-Clamp technique as a high-throughput method to study the electrophysiological properties of mutated connexin hemichannels and the influence of chemical chaperones on restoring connexin function. It automates the traditional patch-clamp technique, enabling rapid and simultaneous recording of multiple cells. Notably, Cx26F161S showed reduced Ca^2+^ sensitivity, but showed no functional rescue by VRT534. Since Ca^2+^ also acts at the pore, this discrepancy suggests that not all pore-level alterations are functionally equivalent, and that VRT534's binding may only restore select gating defects. After treatment with VRT534, a chaperone identified in an earlier study to restore the function of specific Cx26 mutations, we could verify the restoration of channel activity for the Cx26L90P and Cx26R184P mutations. Restoration of channel activity by chemical chaperones has been achieved in cystic fibrosis (CF). The most common mutation is ΔF508 in the cystic fibrosis transmembrane conductance regulator (CFTR) protein. This mutation leads to the misfolding and degradation of CFTR, preventing it from reaching the cell surface to function as a chloride channel (17). Chemical chaperones, such as lumacaftor, have been designed to bind to the misfolded CFTR protein, aiding in its proper folding and trafficking to the cell membrane, thereby restoring its function (28). Lumacaftor and Tezacaftor are now two chemical chaperons clinically approved for the treatment of CF.

Another compound, 4-phenylbutyrate (PBA), has been shown to act as a chemical chaperone by stabilizing the CFTR protein's structure (29). By this stabilization, CFTR can escape degradation in the endoplasmic reticulum and be transported to the cell membrane.

To understand how specific mutations impact the structure and function of Cx26, we developed a structural model that can predict how mutations might alter protein folding, interfere with gap junction assembly, or disrupt hemichannel function. By using this model, we have taken a general approach to probe a preselected chaperone, i.e., VRT534, and identified a possible binding site near the pore, which may be responsible for restoring channel function. The binding of the chemical chaperone VRT534 near the central pore, mediated by its N-terminus, could potentially counteract the effects of the mutation. This interaction might restore the normal function of Cx26, which was blocked by the mutations. This is very likely since the chaperone has already demonstrated the ability to restore hemichannel function (Figure 1C) and (7). In other words, chaperone binding could abolish the mutation's disruptive impact on the protein's activity, although the binding is distant from the mutation site. This has also been shown for other conditions. For example, certain mutations in the gonadotropin-releasing hormone receptor (GnRHR) lead to misfolding and misrouting, causing diseases such as hypogonadotropic hypogonadism. However, specific chemical chaperones can bind to regions of the GnRHR that are not at the mutation site, facilitating correct folding and trafficking to the cell surface, thereby restoring receptor function (30). The use of monomeric models is a limitation. However, current docking algorithms, such as Diff-Dock-L, do not yet support hexameric gap junctions. Future studies utilizing molecular dynamics simulations will be necessary to validate the pore-binding and allosteric effects within the full connexon context. Certain mutations, such as V143A in small heat shock proteins, can impair their oligomerization and chaperone activity, leading to various diseases (31). Suppressor mutations in the N-terminal region, distant from the V143A mutation, can restore oligomerization and function, indicating that interactions at sites away from the original mutation can compensate for structural defects. These examples illustrate how chemical chaperones can bind to specific regions of a protein, which may be located at, close to, or distant from the mutation site, facilitating proper folding and function, similar to the mechanism proposed for connexin hemichannels.

In summary, we present a novel high-throughput and modeling-based strategy to evaluate chemical chaperone candidates for Cx26-related deafness. This approach may facilitate the future design of tailored therapeutics for gap junction disorders. Future progress hinges on overcoming existing delivery and specificity challenges, possibly integrating novel techniques from related fields, such as CRISPR for genetic correction and synthetic biology for system regulation. Emerging insights, potentially elucidated by future studies like ours or previous ones, and continuous research will be essential in unlocking the therapeutic power of chemical chaperones in auditory medicine.

## Data Availability

The original contributions presented in the study are included in the article/supplementary material, further inquiries can be directed to the corresponding author.
